# The Fst/Ldr Family of Type I TA System Toxins: Potential Roles in Stress Response, Metabolism and Pathogenesis

**DOI:** 10.3390/toxins12080474

**Published:** 2020-07-25

**Authors:** Keith Weaver

**Affiliations:** Division of Basic Biomedical Sciences, Sanford School of Medicine, University of South Dakota, Vermillion, SD 57069, USA; kweaver@usd.edu

**Keywords:** type I toxin–antitoxin system, small protein toxin structure, Fst/Ldr family

## Abstract

The *par*_pAD1_ locus was the first type I toxin–antitoxin (TA) system described in Gram-positive bacteria and was later determined to be the founding member of a widely distributed family of plasmid- and chromosomally encoded TA systems. Indeed, homology searches revealed that the toxin component, Fst_pAD1_, is a member of the Fst/Ldr superfamily of peptide toxins found in both Gram-positive and Gram-negative bacteria. Regulation of the Fst and Ldr toxins is distinct in their respective Gram-positive and Gram-negative hosts, but the effects of ectopic over-expression are similar. While, the plasmid versions of these systems appear to play the canonical role of post-segregational killing stability mechanisms, the function of the chromosomal systems remains largely obscure. At least one member of the family has been suggested to play a role in pathogenesis in *Staphylococcus aureus*, while the regulation of several others appear to be tightly integrated with genes involved in sugar metabolism. After a brief discussion of the regulation and function of the foundational *par*_pAD1_ locus, this review will focus on the current information available on potential roles of the chromosomal homologs.

## 1. Introduction

**Figure 1:**
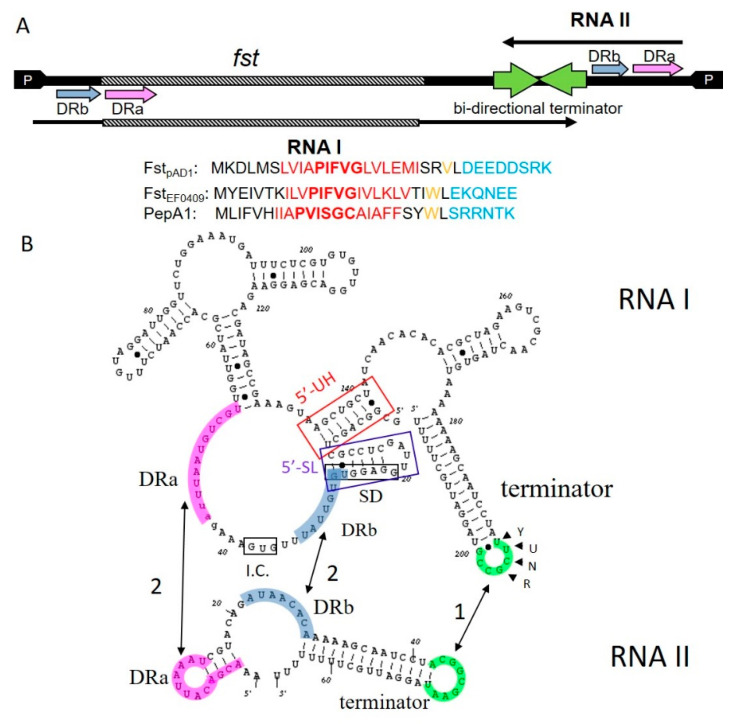
General organization of *par*-like operons and RNA–RNA regulatory interactions as defined in the prototypical *par*_pAD1_ system. (**A**) Genetic organization of *par* and conserved domains within Fst-like proteins. Convergent promoters (arrows labeled P on each end of the DNA map) produce two transcripts (arrows above and below the DNA map): the toxin message, RNA I, and the regulatory RNA, RNA II. RNAs are transcribed in opposite directions across a pair of direct repeats, DRa and DRb, and a bidirectional terminator providing regions of complementarity for interaction. RNA–RNA interactions at DRa and DRb prevent ribosome binding at the toxin coding sequence, *fst*. Shown below the map are the sequences of the two *E. faecalis* Fst paralogs, the plasmid-encoded Fst_pAD1_ and the chromosomally encoded Fst_EF0409_, and the *S. aureus* PepA1 sequence. The conserved hydrophobic domain is shown in red with the PXXXG(C) motif bolded, the charged C-terminal tail is shown in blue and the location of the conserved tryptophan residue is show in orange. Fst_pAD1_ has a valine substitution at this position. (**B**) Map of RNA I-RNA II interaction sites. Interaction is initiated at the loop of the intrinsic terminators (green) stimulated by the YUNR U-turn motif in RNA I. Interaction then spreads to the DRa and DRb sequences that sequester the GUG initiation codon (I.C.) and the Shine–Dalgarno sequence (SD) for Fst. The interaction sites are color coordinated with those shown in the genetic map in Figure 1A. Intramolecular structures within RNA I sequester the ribosome binding site to further delay translation of *fst* (5′-SL) and stabilize the RNA I transcript (5′-UH). These figures were modified from [12].

The *par*_pAD1_ locus of the *Enterococcus faecalis* pheromone-responsive conjugative plasmid pAD1 was the first type I toxin-antitoxin (TA-1) system identified and characterized in Gram-positive bacteria [1,2,3]. It consists of a ~230 nucleotide mRNA, designated RNA I_pAD1_ and encoding the 33 amino acid toxin of the system, Fst_pAD1_, and the ~40 nucleotide sRNA antitoxin, RNA II_pAD1_. The toxin and antitoxin RNAs are transcribed from convergent promoters and share a bidirectional factor-independent transcriptional terminator [3] (Figure 1A). The resultant complementary terminator stem-loops provide the site of initiation of complex formation between toxin and antitoxin RNAs with a U-turn motif in the toxin RNA playing a key role in forming the initial reversible kissing complex [4] (Figure 1B). In addition, the toxin and antitoxin RNAs are transcribed in opposite directions across a pair of DNA direct repeats, DRa and DRb, which provide a second region of complementarity between the RNAs. Interaction in this region suppresses translation of the toxin by sequestering the Fst_pAD1_ translation initiation site [4,5,6]. An intramolecular stem-loop within RNA I_pAD1_, the 5′-SL in Figure 1B, sequesters the Fst_pAD1_ Shine–Dalgarno (SD) sequence until interaction with RNA II_pAD1_ can be completed [7]. Once irreversible interaction is established at all of the complementary regions, RNA I_pAD1_ and RNA II_pAD1_ form a stable complex that facilitates the accumulation of a translationally inactive pool of the toxin message [8]. RNA II_pAD1_ is preferentially degraded from the pool by an unknown mechanism, although an intramolecular “upstream helix” (5′-UH in Figure 1B) sequestering the extreme 5′ end of RNA I_pAD1_ contributes to its differential stability [5,9]. At steady state, the two RNAs are maintained at an approximately equimolar ratio and as long as the plasmid is maintained in the cell, the antitoxin is continually replenished by ongoing transcription [8]. If the plasmid is lost, RNA II_pAD1_ is degraded from the complex pool, Fst_pAD1_ is translated from RNA I_pAD1_, and host cell growth is inhibited [2,10]. Thus, the *par*_pAD1_ locus functions as a typical post-segregational killing (PSK) or addiction module [11]. The salient features of the *par*_pAD1_ locus and the important structural elements of RNA I_pAD1_ and RNA II_pAD1_ are shown in Figure 1.

Exhaustive searches later revealed that *par*-like loci are ubiquitous in the genomes of the Firmicutes (Gram-positive bacteria with a low G + C content) with hundreds of homologs of Fst_pAD1_ identified [12,13,14,15]. While many of these homologs were present on apparent mobile genetic elements (MGE), including phage and pathogenicity islands as well as plasmids, several loci were also identified on core genomes. Expanded examination of the surrounding sequences revealed that all of the structural features present in *par*_pAD1_ were highly conserved, including the convergent promoters, the bidirectional intrinsic terminator, the direct repeats and the regions of intramolecular complementarity in RNA I sequestering the 5′ end and the SD sequence, suggesting that the mechanisms of regulation of toxin expression were broadly conserved. However, not all homologs contained a U-turn motif in any of the predicted loop structures and examination of some of the regulatory details of the chromosomal loci suggest that there is variability in the mechanism of regulation (see below). Since the functions of the non-MGE-located loci must be distinct from *par*_pAD1_, variability in regulatory details are to be expected. In spite of efforts made to characterize other *par*_pAD1_-related loci, the only experimentally verified function of any member of the *par*_pAD1_ family remains the plasmid stabilization function of its founding member.

The purpose of this review is to provide a detailed comparison of the sequence, structure, regulation and effects of the toxins related to Fst_pAD1_. This information will be used to speculate on the possible functions of the toxins in the stress response and pathogenesis of the host organisms.

## 2. Sequence and Structural Comparisons of the Fst/Ldr Family of Small Protein Toxins

Alignment of the Fst_pAD1_ homologs revealed a superfamily signature consisting of a highly conserved tryptophan located between a hydrophobic predicted transmembrane (TM) helix and a highly charged C-terminus [13] (Figure 1A). Interestingly, the conserved tryptophan is replaced by a valine in the Fst_pAD1_ prototype, indicating that it is not essential for function. Surprisingly, this signature was also present in the Ldr family of peptide toxins present in the Gram-negative enterobacterial TA-1 *ldr/rdl* loci [16]. In addition to the defined signature, the majority of the toxins contain a P/D/S/TXXXG(C) motif within the putative transmembrane domain (unpublished observation). The N-terminal residue of the motif varies by clade with the most common PXXXG motif present in *E. faecalis*, *Lactobacillus*, one *Streptococcus pneumoniae* and several *Staphylococcus* clades and the Ldr toxins. The DXXXG motif is present in one *S. pneumoniae* clade, the SXXXG motif in one *Staphylococcus aureus* clade, and the TXXXG motif in several *Staphylococcus* species. The *Listeria* clade is the most divergent of the Fst/Ldr family and has three glycines, one within a PKN(L/I)GF motif which fits in the PXXXG motif clade. Interestingly, all of the *Staphylococcus* isolates, but none of the isolates from other species, contain a cysteine residue at the C-terminal end of the motif and this residue has been postulated to play an important role in the function of this class (see below). While the Ldr and Fst toxins are clearly related, their mechanisms of regulation are distinct; with *ldr/rdl* loci regulated more like the *hok/sok* system of *Escherichia coli* [17].

**Figure 2:**
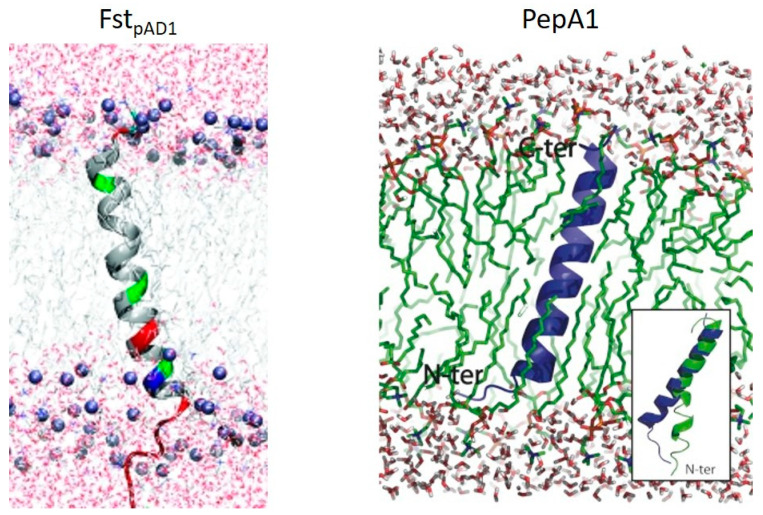
NMR-derived structure of two Fst-family proteins in membrane mimetics. Fst_pAD1_ presents a linear helix postulated to have the N-terminus outside the cell (top) and the C-terminus inside the cell (bottom). Polar amino acids are shown in green, negatively charged residues in red and positively charged residues in blue. Reprinted with permission from [18]. PepA1 forms a similar linear helix spanning the membrane. Membrane interaction appears to straighten a kink produced at the cysteine residue in all staphylococcal Fst-family toxins (inset). Reprinted with permission from [19].

NMR structures of two Fst family members in membrane mimetics have been determined: the Fst_pAD1_ prototype [18] and the PepA1 toxin of the *S. aureus* SprA1-SprA1_AS_ locus [19] (Figure 2). Fst_pAD1_ forms a TM α-helix with the first ~two and last ~seven amino acids protruding. The α-helix structure is maintained at the proline residue in spite of its potential destabilizing effect, but is slightly bent at the glycine residue. The highly charged C-terminal seven amino acids are intrinsically unstructured. Molecular dynamic simulations suggested that the TM α-helix extends from and includes aspartic acid residues at positions 3 and 26. Since the C-terminus contains a stretch of five negatively charged amino acids at the membrane exit point and the external side of Gram-positive membranes is known to be negatively charged, the authors postulated that the C-terminus is located within the cytosol. The TM helix prediction tool TMpred [20] also predicted this orientation. A similar orientation was also proposed for LdrA by molecular modeling [21]. NMR analysis of PepA1 revealed a discontinuous α-helical TM domain with a flexible hinge near the cysteine residue that is unique to the staphylococcal toxins. Molecular dynamic simulations predicted that this α-helix would condense into an extended straight helix when inserted into the cell membrane. Unlike Fst_pAD1_, it was predicted that the C-terminal domain of PepA1 was folded while the N-terminal domain was unstructured. It was also proposed that the arginine-rich C-terminus interacts with the anionic head groups of membrane phospholipids to lock the TM domain in place. PepA1 lacks the multiple acidic amino acids present in the Fst_pAD1_ C-terminus, so the possibility that the two toxins insert into the membrane in opposite orientations must be considered. These structural differences might account for the differences in the behavior of the two toxins. Fst_pAD1_, and also the closely related *fst-Sm* from *Streptococcus mutans*, functions strictly from the inside of the cell and neither lyses bacterial cells nor red blood cells when added extracellularly [18,22,23]. It was proposed that, rather than forming oligomeric pores, the unstructured C-terminus of Fst_pAD1_ might interact with a specific intracellular target [18]. Conversely, PepA1 is capable of lysing both bacterial cells and red blood cells when added extracellularly [24]. It was proposed that the cysteine residue might promote intermolecular disulfide bonds facilitating oligomerization and pore formation [19]. Curiously, PepA2, a *S. aureus* TA-1 toxin 50% identical to PepA1 and containing the same PXXXGC motif, does not lyse bacterial cells extracellularly but is approximately 10X more effective in lysing red blood cells [25]. Thus far, only PepA1 and LdrA have been experimentally determined to be located in the cytoplasmic membrane of their native hosts [19,21].

An extensive mutagenesis survey of Fst_pAD1_ revealed several sequence features affecting toxicity in *E. faecalis* [15]. (1) Maintaining the hydrophobicity of the putative TM domain was essential for toxin function. In most cases isoleucine, leucine and valine could be interchanged for one another but alanine substitutions were not tolerated. (2) The conserved proline and glycine residues in the PXXXG motif were essential for toxicity. (3) The two charged amino acids at the N-terminus (lysine and aspartic acid) could be substituted with either negatively or positively charged, but not uncharged, amino acids without disrupting function. (4) Truncation of the charged C-terminus retained toxicity, although recent data suggests that toxicity is reduced (K. Weaver, unpublished results). In the only other mutagenic analysis of an Fst/Ldr family member published to date, the proline residue of the PXXXG motif of the *Lactobacillus rhamnosus* Lpt toxin was shown to be essential for toxicity when expressed in *E. coli* as for Fst_pAD1_ [26]. Unlike Fst_pAD1_, however, the charged C-terminus was also required.

## 3. Effects of Toxin Over-Expression

As with most TA toxins, the effects of Fst/Ldr superfamily toxins have been determined primarily in toxin over-producing strains. Thus far, overproduction of Fst_pAD1_ [1] and Fst_EF0409_ [27] of *E. faecalis*, PepA1 [19] and PepA2 [25] of *S. aureus*, *fst-Sm* of *S. mutans* [22], and LdrA [21] and LdrD [16] of *E. coli* have all been shown to cause cell death of their native hosts and, in the case of Fst_pAD1_ [15,28], PepA1 [24] and PepA2 [25], several heterologous hosts as well. In addition, the Lpt toxin from a *par* homolog on a *L. rhamnosus* plasmid was demonstrated to be toxic when over-produced in *E. coli* [26]. Disruption of the cell membrane was demonstrated directly for Fst_pAD1_ [23,28], PepA1 [19] and Lpt [26] by DNA staining with membrane impermeant dyes and inferred for LdrA by simultaneous inhibition of macromolecular synthesis (which was also observed for Fst_pAD1_ [23]) and inhibition of ATP production [21]. Lpt was shown to form pores in the outer membrane of *E. coli* cells by atomic force microscopy, but the relevance of this observation to function in a native Gram-positive host without an outer membrane is uncertain [26]. It was also demonstrated that Fst_pAD1_ and nisin, a lantibiotic that disrupts both peptidoglycan biosynthesis and membrane integrity, had synergistic effects on *E. faecalis* cells, strongly supporting a cell envelope effect of Fst_pAD1_ [23]. However, membrane disruption is most likely a secondary effect as time course experiments revealed that nucleoid condensation along with aberrant chromosomal segregation and cell division occurred prior to permeation to DNA staining dyes in both *E. faecalis* and heterologous hosts [28]. Nucleoid condensation was also observed with both LdrD and Lpt [16,26]. A primary role for membrane perturbation has yet to be demonstrated for PepA1 as well, with possible effects on membrane associated functions and/or nucleoid condensation still under consideration [29].

Microarray and RNA-seq analyses revealed the induction of a preponderance of transporters, particularly ATP-utilizing transporters, in response to Fst_pAD1_ expression in *E. faecalis* cells [27,30]. A spontaneous Fst_pAD1_-resistant mutant with a single base change in the *rpoC* gene, encoding the β’ subunit of RNA polymerase, showed reduced transporter induction, suggesting that over-expression of one or more transporters might be responsible for growth inhibition, perhaps by depleting ATP pools. In support of this hypothesis, the inhibition of transporter function with the broad-spectrum translocase inhibitor reserpine had a protective effect against Fst_pAD1_ over-expression [30]. A microarray analysis of cells overproducing LdrD showed the upregulation of genes involved in purine metabolism [16].

Other than the loss of plasmid stability due to the deletion of plasmid-encoded systems, no phenotype has been associated with the deletion of any Fst/Ldr TA-1 system. Plasmid-encoded ectopic over-expression of the complete *S. mutans* Fst-Sm/srSm system led to a dramatic decrease in the number of oxacillin, cefotaxime and vancomycin tolerant persister cells [22]. Since the plasmid-encoded expression of the locus would be expected to proportionately increase both toxin mRNA and antitoxin regulatory RNA, it is not clear how this effect is established and no molecular mechanism has been suggested. Somewhat surprisingly, Michaux et al. were able to construct a mutant in the antitoxin gene of the chromosomal *par*_EF0409_ locus in *E. faecalis* strain V583 [31]. Such a deletion would be expected to be lethal as a result of the loss of the repression of Fst_Ef0409_ expression. However, the transcription of the toxin message from this locus is quite low and, given the intramolecular translational inhibitory structures, expression of the toxin might be below that required to induce cell death even in the absence of the antitoxin. The mutant strain showed increased virulence in a *G. mellonella* larval model and a mouse urinary tract infection model and showed increased resistance to oxidative stress, bile salts, and acidity. The authors suggested that these results implicated the *par*_EF0409_ locus in colonization rather than virulence. Proteomic analysis was also performed to determine the response of the mutant to the absence of the antitoxin RNA. Numerous changes potentially related to the observed increased resistance to stress were identified, but curiously the gene expression changes were distinct from those observed by RNA-seq during mild over-expression of Fst_EF0409_ in *E. faecalis* strain OG1RF [27,31]. Indeed, in one case, the expression of the *arc* operon, the effects were opposite. The reason for these differences is currently unknown, but could relate to differences in toxin expression levels, strain differences, differences between effects on transcription and translation, or the presence of second site suppressor mutations.

Given the various effects of over-production of Fst/Ldr family members, it seems unlikely that they have a generally similar mechanism of action, e.g., forming membrane pores. To examine the degree of variability directly, the transcriptomic response of *E. faecalis* OG1RF cells to Fst_pAD1_ and Fst_EF0409_, plasmid- and chromosomally encoded toxins that presumably have distinct functions, was determined [27]. Results showed substantial differences in response to the two toxins with 113 genes showing higher expression when exposed to Fst_pAD1_ and 90 showing higher expression when exposed to Fst_EF0409_. For example, OG1RF_RS02610, annotated as a copper-translocating P-type ATPase, is induced greater than 100-fold in response to Fst_pAD1_ but not induced significantly by Fst_EF0409_. Conversely, OG1RF_RS01655, encoding an ABC-transporter closely linked genetically to the *par*_EF0409_ locus, is induced 16-fold by Fst_EF0409_ but only eightfold by Fst_pAD1_. These results suggest that, rather than simply poking holes in the membrane, the toxin sequences may be optimized for specific functions. Interestingly, TA-1 toxins in *B. subtilis* unrelated to the Fst/Ldr family have been observed to have subtle effects that similarly bring into question a role in pore formation [32]. Given the small size of the toxins, it should be possible to identify specific amino acids responsible for distinct responses. Such results will pave the way for more detailed structure/function analyses.

At this time, it is unclear how small, membrane-localized proteins could have such disparate effects on nucleoid structure, cell division, stress response and gene expression. It seems unlikely that the proteins act directly with the transcription apparatus, so effects on gene expression are probably an indirect effect of the perturbation of the cell envelope structure or interference with membrane protein function. Discerning the mechanistic aspects of toxin function is a focus of ongoing research.

## 4. Regulation of Toxin Expression

The performance of a post-segregational killing (PSK) function by plasmid-encoded TA-1 systems imposes certain requirements for proper regulation. First, and most obvious, the toxin mRNA must be more stable than the antitoxin so that it can persist in cells that lose the plasmid. Second, the antitoxin must be transcribed at a level high enough to prevent translation from all available active toxin mRNA, but not so high that it allows several generations to pass without killing after plasmid loss. Ideally, the level of antitoxin to translatable toxin mRNA should be close to 1:1. Third, the antitoxin cannot immediately bind and degrade the toxin mRNA, as occurs in most negatively regulated antisense systems, because then no mRNA would remain for translation upon plasmid loss. In the case of the prototypical TA-1 system *E. coli hok/sok*, this is accomplished by the initial adoption of a conformation of *hok* toxin mRNA that can neither be translated nor interact with the *sok* antitoxin, allowing a pool of inactive mRNA to accumulate in the cell. The *hok* mRNA is then slowly degraded from the 3′ end, triggering a conformational change that allows *sok* binding and degradation if the plasmid is retained, or translation if it has been lost (for a review, see [33]). Regulation of *par*_pAD1_ adopts a different strategy with the formation of a mRNA:antitoxin complex that is more stable than either RNA is alone [8]. A pool of the complex is then maintained in plasmid-containing cells, but with continual replacement of the antitoxin RNA removed from the complex. The 5′-SL sequesters the Fst_pAD1_ ribosome binding site to prevent translation of transiently antitoxin-free mRNA [7]. Only after the plasmid is lost is enough antitoxin removed from the complex to free sufficient toxin mRNA for translation. It seems likely that other plasmid-encoded *par* homologs are regulated in a similar manner. One possible exception is the *L. rhamnosus* plasmid-encoded Lpt TA system which shows elevated levels of both toxin and antitoxin RNA under conditions mimicking those that occur during cheese ripening [34]

The regulation of chromosomally encoded TA systems is most likely tailored to their specific functions, so examining the regulatory features of these systems might shed light on their physiological roles. Unlike its plasmid-encoded paralog, the *E. faecalis* antitoxin RNA of chromosomally encoded *par*_EF0409_ is transcribed in large excess over the Fst_EF0409_ toxin mRNA. Indeed, toxin mRNA is barely detectable on Northern blots and is barely above background in qRT-PCR [27,31]. Furthermore, induced expression of the toxin mRNA ectopically reduced levels of the antitoxin RNA suggesting that, unlike in *par*_pAD1_, complex formation destabilizes the antitoxin [27]. Michaux et al. reported that the antitoxin promoter showed threefold decreased activity in the presence of bile salts, a 13-fold decrease in the presence of H_2_O_2_ and a twofold decrease during growth in glycerol as compared to glucose [31]. However, because the antitoxin is produced in such molar excess over toxin mRNA, it seems unlikely that such small stressor-induced decreases in antitoxin RNA would liberate sufficient toxin mRNA for translation. Levels of the antitoxin RNA have also been shown to decrease in the stationary phase, but remain in excess over the toxin message [27]. The plasmid-encoded *par*_pAD1_ and the chromosomally encoded *par*_EF0409_ systems can co-exist in the same cell, apparently without interfering with each other’s function. Thus, RNA II_pAD1_ protects cells from the over-expression of RNA I_pAD1_ but not RNA I_EF0409_ [27]. This is presumably due to variations in the sequence of the DRa and DRb RNA–RNA interaction sites which alignments of *par*-family systems show are poorly conserved.

The regulation of expression of two Fst homologs in *S. aureus*, PepA1 and PepA2, have been examined in detail. As in *par*_EF0409_, SprA1 mRNA, encoding PepA1, is constitutively expressed at low levels and expression of the antitoxin, SprA_AS_, is in large molar excess (35-90-fold) over the toxin mRNA and peaks in mid-exponential phase [24]. PepA1 production was increased by acid stress (twofold) and oxidative stress (threefold) and levels of the antitoxin were decreased by 25% and 50% by those two stresses, respectively. This is consistent with the reported decrease in *par*_EF0409_ antitoxin promoter activity under similar stress conditions [31]. As with Fst_EF0409_, however, it is not clear how a fractional drop in antitoxin production would lead to toxin expression. Nonetheless, the similar expression levels and responses to stress in the two systems from divergent hosts is intriguing.

The second *S. aureus par*-homolog, SprA2/SprA2_AS_, produces both toxin mRNA and regulatory antisense RNA at easily detectable levels, though the relative quantities of the toxin mRNA and antitoxin RNA were not determined [25]. Unlike *par*_EF0409_ but similarly to *par*_pAD1_, overexpression of the toxin mRNA stabilized the antitoxin RNA. This is curious given that the SprA2 system is part of the core genome and therefore likely does not play a PSK role. Antitoxin expression levels were reduced under osmotic shock and stringent conditions, suggesting that it may play a role in stress response, but to different stresses than the SprA1/SprA1_AS_ system.

In summary, while the mechanism and rationale for the regulation of plasmid-encoded PSK systems is well understood, that of the chromosomal systems, as with their functions, remains obscure. Some appear to produce toxin mRNA and antitoxin sRNA at relatively equal levels, while others produce the antitoxin sRNA in high molar excess. In the latter cases, it remains unclear how or when antitoxin levels are reduced sufficiently to allow toxin expression. Further studies on promoter structure and function would certainly be helpful, as would a broader examination of related systems in different species. An examination of this expression under a greater variety of growth conditions, including biofilm and in vivo infection models, would also be helpful.

## 5. Speculations on Function

Except for the well-established role of plasmid-encoded TA systems in maintaining stable inheritance, TA system functions remain largely mysterious and the case of Fst/Ldr systems is no exception. Indeed, it is even tempting to question the canonical PSK function of the plasmid-encoded systems. For example, although the *par*_pAD1_ locus clearly stabilizes heterologous, artificially destabilized plasmids at the expense of host cell growth rate [10], it is reasonable to ask whether this is the most efficient way to stabilize the native pAD1 plasmid. Since the plasmid has a highly efficient conjugation system and enterococci grow in chains, would it not be more efficient to just slow the growth of the plasmid-free segregant and reacquire the plasmid from a neighboring cell in the chain? This possibility certainly raises many questions about how the pheromone-response system required for conjugation would function within an enterococcal chain, but, to the best of our knowledge, no efforts have been directed toward answering these questions.

The functioning of the chromosomally encoded Fst/Ldr systems is even more speculative. As is common for TA systems in general, the deletion of the Fst/Ldr TA systems has not been associated with any phenotypic effect on host cells; so either their functions are redundant with other bacterial genes or conditions have not yet been found under which their function can be observed. As described above, overexpression of the toxins has been demonstrated to have numerous effects on the host cells, but it is unclear whether such high expression levels are ever obtained under normal physiological conditions. A variety of stress conditions have been shown to modestly reduce the expression of antitoxin RNA, which could conceivably increase toxin levels and affect growth, but this observation comes with a number of caveats. First, in the SprA1 and Fst_EF0409_ cases, the antitoxin is produced in such excess over the toxin mRNA levels that it is hard to see how modest changes in antitoxin levels could lead to sufficient toxin expression to have an effect [24,31]. Increased toxin expression was observed in the SprA1 system, but only when the entire locus was present ectopically on a multicopy plasmid [19]. With Fst_EF0409_, improved growth was observed in an antitoxin knockout under the same conditions that showed reduced antitoxin expression in the wild type, which provides some circumstantial evidence that the system may be involved in stress survival [31]. However, genomic sequencing should be performed to ensure that this mutant does not have compensatory mutations that allow the cell to grow in the presence of the toxin. The SprA2 antitoxin RNA also appears to be regulated by stress conditions [25], but whether this affects toxin expression has not been addressed. Overall, the evidence supporting a role for these TA loci in stress response is not strong.

The *S. aureus* SprA1-SprA1_AS_ system is located on a pathogenicity island [24] and therefore could conceivably function as a PSK to ensure maintenance of that MGE. However, the vast excess of antitoxin RNA under the conditions examined thus far makes such a role unlikely. Both PepA1 and PepA2 are cytolytic for human cells and therefore could be considered as virulence factors during *S. aureus* infection [24,25]. However, no evidence from animal models has been presented supporting such a role. Furthermore, it is not clear how the peptide toxin would be released from the membranes of the producing bacterium to attack red blood cells. Synthetic SprA1 also lyses Gram-positive and Gram-negative bacterial cells [24], but, again, problems with release from the producing cell’s membrane would limit its usefulness in microbial competition, though it does provide potential as an antibacterial agent [35].

**Figure 3:**
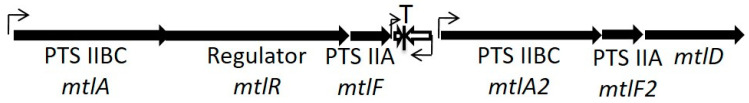
Genetic context of *par*_EF0409_. The “T” marks the position of the bidirectional terminator shown in Figure 1A with the shorter white arrow on the left depicting the position of RNA II and the longer white arrow on the right depicting RNA I. The locus is situated between two paralogous mannitol-family phosphotransferase systems. The upstream operon includes *mtlR* encoding a putative positive regulator of the downstream operon.

Circumstantial evidence based on gene location suggests that some of the chromosomal *par* homologs may be integrated with core metabolic functions. For example, *par*_EF0409_ is located in all sequenced *E. faecalis* strains between two paralogous mannitol class phosphotransferase systems [15] (see Figure 3). Our unpublished results suggest that the downstream operon plays an essential role in mannitol transport, while the upstream operon plays a regulatory role, particularly the *mtlR* gene, which is homologous to mannitol regulators in other Gram-positive bacteria [36]. Interestingly, there is no transcriptional terminator between the *mtlF* gene and the antitoxin RNA, suggesting that readthrough transcription could modulate expression of the toxin by altering antitoxin levels. We have also observed that mannitol-grown cells are approximately 10-fold more sensitive to Fst_EF0409_ than glucose-grown cells. Finally, a mutation of a genetically linked ABC transporter increased toxicity in both glucose and mannitol, suggesting that this transporter was important for recovery from toxin expression. How exactly Fst_EF0409_ might fit into the regulation of mannitol transport and/or metabolism is not clear, but it is worth noting that small peptides have been shown to directly modulate the transport of other sugars [37]. Alternatively, we also note that there are significant regions of DNA sequence homology between the *mtlA* and *mtlA2* genes that could result in the recombination and excision of the intervening DNA. The *par*_EF0409_ locus could conceivably perform a PSK role to keep the excised circle of DNA from being lost, although the ratio of antitoxin to toxin mRNA would not be ideal for this function. Several other chromosomally encoded *par* homologs show a similarly integrative association with core metabolism genes, but little is known about how these systems are regulated and function [14,15].

## 6. Conclusions

The Fst/Ldr proteins are a widespread family of apparently membrane active peptides regulated by small RNAs in TA-1 systems. The Fst subfamily is present in the Firmicutes, including in pathogenic species of *Staphylococcus*, *Streptococcus*, *Enterococcus* and *Listeria* as well as important commensal species of *Clostridia* and *Lactobacillus*. The Ldr subfamily is present in the enterobacteria, including pathogenic species of *Escherichia*, *Shigella*, and *Salmonella*. While the toxins of the two subfamilies clearly share conserved motifs, their regulatory mechanisms are distinct, and it is unclear whether they share a common ancestor or evolved convergently. Their taxonomic coherence with the phylogenetic trees of their host bacterial species suggest that they have not been spread broadly by horizontal genetic transfer despite their presence on MGE [13].

Although widespread in numerous bacterial pathogens, the demonstration of a specific role in pathogenesis for the Fst/Ldr family has been elusive. The prototype Fst-encoding TA-1 system, *par*_pAD1_, has been demonstrated to perform a stabilization function for its host plasmid and related systems are likely to perform similar functions for other MGE. The locus is highly conserved on pheromone-responsive conjugative plasmids of *E. faecalis*, which are known to encode both antibiotic resistance and virulence factors [38,39,40], and therefore likely plays a critical role in the maintenance and dissemination of these determinants in this important pathogen. Other putative roles in pathogenesis are more speculative. Limited toxin expression could conceivably function to slow bacterial growth and lead to the accumulation of persisters, but no evidence of such an effect has been observed and some evidence actually contradicts such a role [22]. Expression analysis suggests that several of the Fst-related toxins may be involved in stress response [24,25,31], but the mechanisms have yet to be elucidated. An *E. faecalis* strain over-producing the Fst_EF0409_ was found to increase virulence in two model systems [31], but the conditions that would lead to such levels of toxin production have not been defined. Synthetic derivatives of two *S. aureus* Fst-related toxins have been demonstrated to be cytolytic for both human and bacterial cells [24,25], but whether these toxins are actually secreted from natural producers has yet to be demonstrated. Circumstantial evidence suggests that some related loci are integrated with basic sugar metabolism [14,15], but evidence for a specific role is lacking.

Thus, like most other TA system toxins, the role of the Fst/Ldr toxins in pathogenesis, and indeed in metabolism in general, remains frustratingly obscure. A clear demonstration of function remains tantalizingly just beyond our reach. Once a function has been defined for a specific system, it will be important not to jump to the conclusion that all of the related systems perform the same function. The possibility that their functions may be tailored to the needs of specific species must always be considered. Clearly, more work is justified and required to identify the role(s) of these ubiquitous systems.
